# Perceived Stress Scores among Saudi Students Entering Universities: A Prospective Study during the First Year of University Life

**DOI:** 10.3390/ijerph110403972

**Published:** 2014-04-10

**Authors:** Nasser M. Al-Daghri, Abdulaziz Al-Othman, Abdulmajeed Albanyan, Omar S. Al-Attas, Majed S. Alokail, Shaun Sabico, George P. Chrousos

**Affiliations:** 1 Prince Mutaib Chair for Biomarkers of Osteoporosis, Biochemistry Department, King Saud University, Riyadh 11451, Kingdom of Saudi Arabia; E-Mails: amothman@ksu.edu.sa (A.A.-O.); omrattas@ksu.edu.sa (O.S.A.-A.); msa@yahoo.co.uk (M.S.A.); eaglescout01@yahoo.com (S.S.); 2 Biomarkers Research Program, Biochemistry Department, College of Science, King Saud University, Riyadh 11451, Kingdom of Saudi Arabia; E-Mail: chrousos@gmail.com; 3 Center of Excellence in Biotechnology Research Center, King Saud University, Riyadh 11451, Kingdom of Saudi Arabia; 4 College of Applied Medical Sciences, King Saud University, Riyadh 11451, Kingdom of Saudi Arabia; 5 Preparatory Year Program, King Saud University, Riyadh 11451, Kingdom of Saudi Arabia; E-Mail: aalbanyan1@ksu.edu.sa; 6 First Department of Pediatrics, Athens University Medical School, Athens 11527, Greece

**Keywords:** stress, college life, Saudi students

## Abstract

In this prospective study we wanted to determine whether perceived stress over time among students in the Preparatory Year of King Saud University (KSU) predisposes them to cardiometabolic abnormalities. A total of 110 apparently healthy Saudi students (35 men and 75 women enrolled during the 2010−2011 academic year) were included. Perceived stress was determined at baseline and 1 year later. Anthropometrics were obtained and morning fasting serum glucose, lipid profile and cortisol were measured at both times. Perceived stress was noted among 48.2% of subjects at baseline and was not significantly different after follow-up, with 45.4% scoring high. In men, the prevalence of perceived stress was 48.6% at baseline (13 out of 35) and 37.1% at follow-up (13 out of 35), while in women it was 48% at baseline and 49.3% at follow-up. Interestingly, significant improvements in the blood pressure and lipid profiles, with the exception of HDL-cholesterol, were observed in both men and women, while fasting glucose also improved in women. Serum cortisol was inversely associated to fasting glucose, and total- and LDL-cholesterol (*p*-values 0.007, 0.04 and 0.04, respectively). These data are opposite to findings in students entering Western universities, in whom increasing stress and a deteriorating cardiometabolic profile have been repeatedly noted. Perceived stress and morning cortisol levels among students of the Preparatory Year in KSU remained constant for both genders over time, yet an improved cardiometabolic profile was observed, suggesting good adaptation among our pre-college students in their first year of university life.

## 1. Introduction

There is accumulating evidence that college students have alarming rates of psychological distress [1,2]. Improper coping with stress may lead to self-destructive and aggressive behaviors as dark as suicide [3,4]. Consequently, chronic exposure to stress can significantly alter the physiology of the mind and body, leading to several stress-related diseases [5]. Children and adolescents are more sensitive to the effects of chronic stress, which places them at an increased risk of developing chronic diseases if they are not guided appropriately on coping with and adapting to stressors [6].

There have been several studies indicating that adolescents who are about to begin their university lives are at high risk for abnormal weight gain secondary to tangible environmental stimuli and disordered eating in men, but more so, in women [7,8]. Several protective factors include regular exercise and good relationships with parents in men and positive relationships with parents in women [9]. In both genders, changes in eating habits are considered an independent predictor of weight gain [10].

In this prospective study, our aim was to monitor the perceived stress of incoming students entering and completing their Preparatory Year of KSU, using a well-known tool in assessing global measure of perceived stress that has been used and validated in several studies [11,12,13,14,15,16], in an attempt to understand whether perceived stress changes among Arab young adult students translate to altered metabolic patterns during this period.

## 2. Experimental Section

### 2.1. Subjects

In this prospective study, a total of 110 apparently healthy young adult Saudi students (35 men and 75 women during the 2010−2011 academic year) were included. The study was conducted in the Preparatory Year (PY) Building of King Saud University, Riyadh, Saudi Arabia, and was done in collaboration with the PY dean and with the support of PY faculty members, most notably the physical education instructors who helped recruit students in their respective classes. The study is part of a larger cross-sectional research project of the PY involving 1,878 Saudi students (1,112 males and 766 females). Participating students were rewarded with minor items (shirts and snacks) as a token of appreciation, and those who consented to participate in the follow-up study were rewarded with additional credits both for course and extra-curricular activities, as deemed appropriate by the assigned faculty member.

### 2.2. Design Questionnaire

Students answered a pre-designed questionnaire that was developed, pre-tested and validated in a pilot study. The questionnaire was based on a previously published article and has an internal consistency of 0.85 (Cronbach α co-efficient) and test-retest reliability during a short retest interval (several days) of 0.85 [17]. For the present study, the questionnaire had an internal consistency of 0.65 (Cronbach α co-efficient) over-all. The questionnaire has several parts including: socio-demographic data, medical history, physical activity, sleep assessment, and a perceived stress test [16,17]. Perceived stress test scores are obtained by reversing the scores on four positive items, with a possible range of scores from 0 to 56. PSS scores were then dichotomized, with a score of 28 and above labeled as “stressed” and 27 below as “not stressed” [17]. Ethics approval was obtained from the College of Medical Research Center (CMRC), King Saud University, Riyadh, Saudi Arabia. Written and informed consents were required from participants prior to inclusion. Approval from the Dean of the Preparatory Year was also sought prior to commencement of studies.

### 2.3. Inclusion and Exclusion Criteria

Only consenting students with unremarkable medical history from the Preparatory Year were included in the study. Out of the original 1,878 students who participated on the cross-sectional survey, only 110 consented to be followed-up for one year. The large drop-out was secondary to the non-mandatory nature of participation in the present study, which was not the case in the cross-sectional survey where all students were required by school administration to participate. Students with underlying acute and chronic medical conditions that warrant immediate attention (e.g., asthma), and students who were disabled were excluded from the follow-up study.

### 2.4. Anthropometrics

Participating students had their anthropometrics assessed wearing light clothing in an isolated room by the assigned school nurse. Weight was recorded to the nearest 0.2 kg using an international standard scale (Digital Person Scale, ADAM Equipment Inc., USA); height to the nearest 0.5 cm using the same scale. BMI was calculated as kg/m^2^, and classified as lean or obese, depending on BMI for age and gender for subjects below 18 years old. Waist and hip circumferences were measured by non-stretchable tape measure, and recorded to the nearest 0.1cm. Body fat composition was also assessed by Body Fat Analyzer (Biospace Co., Ltd., Seoul, South Korea). All measurements were administered by well-trained BRP research assistants. All measurements were repeated after 1 year.

### 2.5. Biochemical Assessment

Blood was collected at baseline and one year later. Blood was withdrawn in the morning after an overnight fast (>10 h) and collected in non-heparinized test tubes by an assigned physician. Fasting serum glucose and lipid profile (HDL-, LDL-, total cholesterol and triglycerides) were measured using routine laboratory procedures (Konelab, city, Finland). Levels of cortisol in serum was measured by electrochemiluminescence immunoassay (ECLIA) on a Roche Elecsys 411 system (Roche Diagnosis Elecsys, Indianapolis, IN, USA). This method can measure the concentrations of cortisol in the range of 0.5−1,750 nmol/L and has analytical sensitivity of 0.5 nmol/L. The intra- and inter-assay variation for cortisol were 3.8% and 4.2%, respectively. All biochemical estimations and storage of samples were carried out in the Biomarker Research Program (BRP) of King Saud University, Riyadh, Kingdom of Saudi Arabia.

### 2.6. Data Analyses

Sample size calculation was done based on the mean cortisol differences across follow-up which is the variable of interest (see Table 1 for actual cortisol values and standard devitaion). Based on the given means, a sample size of *N* = 72 has an actual power of 0.80 with α = 0.05 and an effect size of 0.34 (critical *t* = 1.99). The present sample size of *N* = 110 has a power of 0.90 to detect difference at α = 0.03.

**Table 1:** General Demographic and Clinical Characteristics of the Subjects.

	2011	2012
N	110	110
Gender (M/F)	35/75	
Perceived Stress Score	27.3 ± 5.0	27.3 ± 5.5
Sleep Quality (% Continuous)	55.8	
Sleep Duration (Hours)	7.3 ± 1.8	
Physical Activity (% Yes)	52.6	
Age (years)	19.1 ± 0.95	
BMI (kg/m^2^)	24.2 ± 6.3	24.6 ± 6.8
Waist (cm)	75.7 ± 17.6	78.8 ± 16.3
Hips (cm)	99.7 ± 14.4	100.6 ± 15.4
Systolic BP (mmHg)	116.3 ± 11.7	107.1 ± 11.1 ***
Diastolic BP (mmHg)	73.7 ± 6.8	69.3 ± 7.5 ***
Glucose (mmol/L)	4.9 ± 0.67	4.7 ± 0.71 ***
Triglycerides (mmol/L)	1.02 ± 0.60	0.98 ± 0.47
Total Cholesterol (mmol/L)	4.3 ± 0.93	3.5 ± 0.56 ***
HDL-Cholesterol (mmol/L)	1.23 ± 0.34	0.99 ± 0.20 ***
LDL-Cholesterol (mmol/L)	2.8 ± 0.81	2.3 ± 0.56 *
Cortisol (nmol/L)	425.6 ± 19.0	420.0 ± 17.1

Note: Data presented as mean ± SD; P-value significant at * *p* < 0.05, *** *p* < 0.001

Data were analyzed using the SPSS (SPSS version 16.5, Chicago, IL, USA). Continuous variables were presented as mean ± standard deviation. Cortisol values were log transformed prior to analysis because it was not normally distributed as compared to other variables. Paired Student t-test was done to compare baseline and follow-up visits. Spearman correlation was done to assess significant associations with variables of interest at baseline and follow-up. Significance was set at *p* < 0.05.

## 3. Results and Discussion

Table 1 shows the general characteristics of the subjects studied. Perceived stress was noted among 48.2% of subjects (53 out of 110) at baseline and 45.4% (50 out of 100) at follow-up. In males, the prevalence of perceived stress was 48.6% at baseline (13 out of 35) and 37.1% at follow-up (13 out of 35), while in females it was 48% at baseline and 49.3% after follow-up (not included in the Table). Thus, no changes were noted in the perceived stress scores over the preparatory year. Significant improvements in the systolic and diastolic blood pressure, fasting serum glucose and total cholesterol were observed throughout (*p*-values < 0.001, <0.001, <0.001, and <0.001, respectively. There was a significant decrease in LDL-cholesterol (<0.001), as well as HDL-cholesterol levels (<0.001). No further significant associations were identified among women.

Subjects were then analyzed according to gender. In men, there was a significant increase in waist circumference and glucose levels over baseline (*p*-values 0.03 and 0.02, respectively). Overall, there was a significant decrease in the levels of total, LDL- and HDL-cholesterol levels (*p*-values 0.005, 0.002 and 0.05, respectively) in the follow-up as compared to baseline. In women, there was a significant improvement in the blood pressure, glucose and lipid profile, with the exception of HDL-cholesterol (see Table 2). In both genders, there were no significant differences in the perceived stress scores at the first and follow-up evaluation.

**Table 2:** General Demographic and Clinical Characteristics of Subjects According to Gender.

	Men		Women	
	2011	2012	2011	2012
Perceived Stress Score	27.6 ± 5.4	26.3 ± 4.4	27.2 ± 4.8	27.8 ± 5.9
N	35	35	75	75
Age (years)	18.8 ± 0.75		19.7 ± 1.0	
BMI (kg/m^2^)	24.9 ± 5.8	25.8 ± 6.3	24.0 ± 6.5	24.3 ± 6.3
Waist (cm)	81.6 ± 18.4	87.6 ± 18.4 *	74.7 ± 12.2	73.4 ± 11.2
Hips (cm)	99.9 ± 17.9	103.6 ± 19.1	99.6 ± 12.2	99.1 ± 12.9
Systolic BP (mmHg)	112.3 ± 10.3	109.6 ± 10.1	117.3 ± 11.7	106.3 ± 11.1 **
Diastolic BP (mmHg)	73.2 ± 6.1	70.3 ± 6.5	74.1 ± 6.8	68.5 ± 7.5 **
Glucose (mmol/L)	4.8 ± 0.94	5.2 ± 0.74 *	4.9 ± 0.43	4.3 ± 0.42 **
Triglycerides (mmol/L)	1.2 ± 0.66	1.2 ± 0.48	0.91 ± 0.54	0.86 ± 0.43
Total Cholesterol (mmol/L)	4.2 ± 1.1	3.5 ± 0.63 **	4.3 ± 0.74	3.4 ± 0.51 **
HDL-Cholesterol (mmol/L)	1.0 ± 0.30	0.93 ± 0.18 *	1.4 ± 0.31	1.0 ± 0.20 **
LDL-Cholesterol (mmol/L)	2.9 ± 0.95	2.4 ± 0.61 **	2.8 ± 0.73	2.3 ± 0.53 **
Cortisol (nmol/L)	383.3 ± 16.8	418.6 ± 22.0	485.3 ± 18.5	421.0 ± 17.6

Note: Data presented as mean ± SD; * denotes significance at *p* < 0.05, ** denotes significance at *p* < 0.001

Lastly, cortisol levels were significantly and positively correlated with glucose (R = 0.29; *p* = 0.003), triglycerides (R = 0.24; *p* = 0.014) and LDL-cholesterol (R = 0.203; *p* = 0.04) (see Table 3). No further significant associations were identified between cortisol levels and the other parameters measured at baseline, including perceived stress.

**Table 3:** Spearman’s Correlation between Serum Cortisol (nmol/L) with Cardiometabolic Parameters according to Follow-Up.

Parameter	Baseline	1 year Follow-Up
BMI (kg/m^2^)	−0.06	−0.12
Waist (cm)	−0.13	0.07
Hips (cm)	−0.10	−0.18
Systolic BP (mmHg)	0.03	0.08
Diastolic BP (mmHg)	0.06	−0.008
Glucose (mmol/L)	0.07	**0.29** **
Triglycerides (mmol/L)	0.006	**0.24** *
Total Cholesterol (mmol/L)	0.02	0.18
HDL-Cholesterol (mmol/L)	0.05	−0.07
LDL-Cholesterol (mmol/L)	0.06	**0.20** *

Note: Data presented as coefficient (R); * denotes significance at 0.05 level; ** denotes significance at 0.01 level; *p*-value significant at < 0.05.

The main findings in this prospective study is that students entering the preparatory year were able to “adapt” to the stressful environment of pre-college, as evidenced by the favorable metabolic profile at the completion of their first year, and the non-significant change of stress levels despite being exposed to added stress of university life. It has been consistently documented that the environment provided by universities and other academic institutions can be stressful to students and this can contribute to altered metabolic and behavioral patterns [8,18,19]. Findings from previous observations contradict the present study. Among the cardiometabolic factors studied, there was an improvement in blood pressure and cholesterol levels among students. However, several factors also started to deteriorate, such as fasting glucose and HDL-cholesterol, suggesting that maybe only a select number of students are less adaptive with a greater predisposition to stress-induced metabolic changes.

The significant associations of fasting cortisol between glucose and the select lipid profile are worthy of mention as they confirm previous findings. Indeed, long-term, even mildly elevated cortisol levels can have detrimental effects on the beta cells of the pancreas and may significantly increase fat accumulation, diminish skeletal muscle mass and alter carbohydrate and lipid metabolism [5,6,20,21,22]. Generally, in chronically stressed individuals there is a flattening of the circadian cortisol curve with lower morning peak levels and higher evening zenith levels than non-stressed ones [23,24,25].

The presence of stress and the activation of the stress system in both adults and children is known to affect a variety of endocrine, metabolic, behavioral and cardiovascular functions, which, if persistent, can lead to a variety of diseases later on in life [6]. Children and adolescents, in particular, are sensitive to chronic alterations in cortisol secretion, which can greatly affect cognitive and emotional development [21]. One explanation for the lack of conformity of findings was the timing of blood collection and cortisol measurements. Cortisol follows a circadian rhythm with zenith levels at a 2−4 hour window after waking and then decrease to a nadir at a 2−4 hour window around sleeping time [26]. We cannot ascertain whether there were changes in sleep patterns, and did not obtain evening cortisol levels, as the latter would be necessary to confirm the presence of diurnal variations and/or disruptions in the circadian rhythm. This limits the study since alterations in sleep and the cortisol circadian rhythm are significantly linked to stress [27,28]. The significant waist circumference gain in males as compared to females confirms the study of Ernersson and colleagues in that young males are more likely to accumulate fat mass in the abdominal region than females with fast food consumption and limitation of physical activity [29], while the lack of significant gain in BMI might be partially attributed to the continuing longitudinal growth in some, if not all, of the male subjects and/or may simply indicate redistribution of body compartments without changes in height or body weight.

In the present study, our young adult subjects were subjected to stress secondary to a change in environment and social life in the year of pre-college education. Despite this exposure, there were no significant differences in the perceived stress score one year after baseline collection in both men and women, as well as no significant changes in the cortisol levels, suggesting that probably the incoming students were able to adapt positively with the change in environment. Another plausible explanation might be the social environment of our students is not as stressful as that of Western academic institutions. The Preparatory Year has only 4 programs that include English, computer, basic sciences and self-development skills, and each student is to develop these skills on different tracks, depending on the career they wish to pursue (medicine, engineering and humanities) [30]. Religious practice is very much evident and a daily part of our students’ activities, promoting positive spiritual growth and outlook in life. Spiritual health has been negatively associated with stress and depressive tendencies and positively associated with health-promoting behaviors among students from other ethnic groups [31,32]. Lastly, prohibition of alcohol use and a structured daily life program, including regular times of working, eating and sleeping may have contributed to the mostly favorable cardiometabolic changes in our preparatory year students. This adaptation, while only true for the preparatory year students, is acutely favorable, considering Arab families have a strong genetic predisposition to abnormal metabolic conditions later on in life [33].

The study has several limitations. The small sample size of the students followed up might contribute to type 2 errors in associations that would have been avoided had the size been bigger. Stress was not measured before college entry, which may explain the high % percentage of perceived stress at baseline which remained the same after follow-up. Furthermore, several variables that include physical activity, diet and changes in sleep patterns have not been taken into consideration in the analysis.

## 4. Conclusions

In summary, perceived stress and cortisol levels among students of the Preparatory Year over time remained constant in both genders. Yet an improved cardiometabolic profile was observed during this time period, suggesting an increase in coping or adaptation regardless of the stress level among our students. This is different from data in Western institutions of higher learning, in which perceived stress and cardiometabolic risk indices are noted to increase in the first college year. Further studies are required to evaluate whether this observed reduction in cardiometabolic risk continues in Saudi students during their subsequent years in the university.
